# Selective Laser Trabeculoplasty vs. Fixed Combinations with Timolol in Practice: A Replacement Study in Primary Open Angle Glaucoma

**DOI:** 10.4274/tjo.87300

**Published:** 2017-08-15

**Authors:** Ali Kutlay Tufan, İsmail Umut Onur, Fadime Ulviye Yiğit, Ahmet Ağaçhan, Şenay Aşık Nacaroğlu

**Affiliations:** 1 Health Sciences University, Bakırköy Dr. Sadi Konuk Training and Research Hospital, Ophthalmology Clinic, İstanbul, Turkey

**Keywords:** Fixed combination antiglaucoma medications, intraocular pressure reduction rate, primary open-angle glaucoma, selective laser trabeculoplasty

## Abstract

**Objectives:**

To evaluate the potential of selective laser trabeculoplasty (SLT) in two arms (360˚ vs. 180˚) as a replacement for fixed combinations (FCs) with timolol in primary open angle glaucoma over 6 months.

**Materials and Methods:**

Of 40 patients in a prospective, comparative, interventional case series, 18 eyes and 22 eyes were randomized to SLT 180º and SLT 360º groups, respectively, along with 40 fellow-control eyes. FC with timolol was discontinued on the day of treatment for the eye to be operated on, while ongoing therapy was not interrupted for the contralateral eye. Eyes were examined for intraocular pressure (IOP) elevation 1 hour and 1 day after SLT. The follow-up visits were then scheduled for 1 week, 1 month, 3 months, and 6 months after, during the which the IOP of both eyes and any possible complications were evaluated.

**Results:**

There were no statistically significant differences in mean IOPs through 6 months among the groups with exception of postlaser 1 hour and postlaser 1 day (p<0.001 and p=0.010, respectively). Multiple comparison analysis showed significantly higher IOP in both SLT 180º and SLT 360º subgroups compared to their controls at postlaser 1 hour (p=0.007, p<0.001) but significantly lower IOP only in SLT 360º subgroup compared to the controls at postlaser day 1 (p=0.013).

**Conclusion:**

SLT offers promising potential as a substitute equivalent to efficacy of FCs with timolol. However, SLT 360˚ may not achieve additional IOP reduction.

## INTRODUCTION

Glaucoma is the second leading cause of blindness worldwide and 74% of the patients have primary open angle glaucoma (POAG).^1^ The current treatment paradigm aims to decrease intraocular pressure (IOP), initially with pharmacotherapy, performing laser trabeculoplasty as the second step, and resorting to incisional surgery as a final option.^2^ However, medical treatment has some inherent drawbacks such as nonadherence, tachyphylaxis associated with chronic administration, and the financial burden imposed by high pharmaceutical costs.^3,4,5,6^ As a consequence, one eye goes blind in 27% of patients receiving medical treatment for 20 years.^7^. In order to maximize patient adherence and quality of life, several fixed combinations (FCs) of commonly used IOP-lowering medications have been developed recently, which include the topical beta-blocker 0.5% timolol combined with a prostaglandin analogue, alpha-adrenoceptor agonist, or topical carbonic anhydrase inhibitor.^8,9^ A relatively recent meta-analysis evaluated 41 eligible randomized clinical trials on 53 arms investigating the efficacy of 6 FCs after medicine-free washout periods and reported the relative reductions for mean diurnal IOP as 34.9% for travoprost/timolol, 34.3% for bimatoprost/timolol, 33.9% for latanoprost/timolol, 32.7% for brinzolamide/timolol, 29.9% for dorzolamide/timolol, and 28.1% for brimonidine/timolol. However, from the statistical standpoint, the meta-analysis concluded that only latanoprost/timolol and travoprost/timolol are likely to achieve better IOP reduction among these combinations, and the comparisons mostly remain within the non-inferiority margin.^10^

Selective laser trabeculoplasty (SLT), described by Latina and De Leon^11^, is a relatively novel therapeutic approach reported to be equally efficacious as both a first-line medication and argon laser trabeculoplasty (ALT).^12^ SLT requires less than 1% of the energy used in ALT and thereby causes minimal thermal burn to the trabecular meshwork.^13,14^ Since the IOP-lowering mechanism of SLT is associated with biochemical and cellular pathways rather than mechanical or thermal effects^15,16^, it is considered to be possible to repeat the procedure over time, which enhances the potential cost-saving feature as opposed to medication.^17^ With respect to therapeutic efficiency, there are several studies reporting relative IOP reductions from baseline ranging between 26.4% and 35.1%,^2,18,19,20,21,22,23,24^ which are consistent with the IOP reduction rates of the 6 FCs mentioned above. However, to our knowledge only one study evaluating SLT as a replacement for medical therapy reported reduction in number of antiglaucoma medications by a mean of 2.0 at 6 months [95% confidence interval (CI) 1.8-2.3] while keeping the IOP within the target range.^25^ As a result, SLT theoretically seems to reduce IOP comparable to FCs, which it may substitute for in practice.

Based on the assumption that all FCs reduce IOP within a non-inferiority margin, in this study we aimed to evaluate the potential of SLT as a replacement for FCs by comparing reduction in IOP over 6 months for POAG. We further compared the efficacy of SLT 360° and SLT 180° applications.

## MATERIALS AND METHODS

This study was designed as a prospective, comparative, interventional case series and was conducted between December 2012 and June 2013. After obtaining the institutional ethics committee approval [Health Sciences University, Bakırköy Dr. Sadi Konuk Training and Research Hospital (2012-116)], patients’ charts in the glaucoma unit of our tertiary referral hospital were reviewed and the following criteria were sought for recruitment:

a) Presence of bilateral POAG,

b) Both eyes receiving the same antiglaucoma medications and dosing which currently included an FC of 0.5% timolol maleate,

c) IOP of both eyes ≤23 mmHg (average of the last 3 measurements) and equal (difference between IOP of both eyes ≤2 mmHg in the last 3 measurements).

On chart reviews, glaucoma was confirmed on the basis of glaucomatous disc damage (vertical cupping, diffuse and focal neural rim thinning) with at least 2 reliable visual field (VF) tests (Humphrey Field Analyzer, Swedish Interactive Threshold Algorithm 24.2 test, Carl Zeiss Meditec, Dublin, CA, USA) which denote fixation losses <20% along with false positives and negatives <30%. Scotomas of 3 contiguous points at the level of 5% on the pattern deviation plot were sought on successive VFs. Alternatively, spectral domain-optical coherence tomography were referred to for at least 1 sector of peripapillary retinal nerve fiber layer (pRNFL) thinning at the level of 1% or 2 contiguous sectors of pRNFL thinning at the level of 5% on the temporal-superior-nasal-inferior-temporal plot conforming to disc changes at least on 2 occasions when reliable VFs were absent (RNFL 3.45 protocol, RTVue-100 OCT, Optovue Inc, Fremont, CA, USA). Thus, 44 patients meeting the abovementioned criteria were then interviewed and informed about the study and asked for verbal and written consent on a voluntary basis. Four of these patients were later excluded due to cataract surgery (1 patient), nonadherence to antiglaucoma medication use (2 patients) and loss to follow-up (1 patient). The study was conducted in accordance with the Declaration of Helsinki.

One eye of the patients was randomly selected for laser therapy and included in the intervention group while ongoing medical treatment was continued on the contralateral eye, which was included in the control group. The eyes in the intervention group were further randomized into SLT 180° or SLT 360° laser subgroups (by U.O.). Prior to laser therapy, both eyes underwent comprehensive ophthalmic examination in which medical and ophthalmic history, refraction, best corrected visual acuity, slit lamp biomicroscopy, IOP (Goldmann applanation tonometry) and fundoscopy were included in order to confirm the records on the charts. Gonioscopy was carried out using a 3 mirror lens (Design-OG3M-10, Ocular, Bellevue, WA, USA) to confirm angles of the eyes were open in 3-4 quadrants (Shaffer grades of 3-4) (by K.T.).

Patients with a history of previous intraocular operations or laser procedures, pseudoexfoliation or pigmentary glaucoma, advanced glaucoma (vertical cup/disc ratio >0.8) were excluded. Eyes with signs of corneal and/or lens abnormalities that might preclude precise tonometry or visualization of the cup and optic disc were also excluded.

Q-switched, frequency-doubled Nd:YAG laser of 532 nm wavelength (Selecta 2, Lumenis, Coherent, Inc., Palo Alto, CA, USA) was used for treatment. The pulse duration and spot size were 3 ns and 400 µm, respectively. Following topical anesthesia with 0.5% proparacaine hydrochloride, pigmented trabecular meshwork was targeted and non-overlapping laser spots were evenly placed on either the inferior 180º or the entire 360º of the trabecular meshwork with a specifically designed SLT gonio lens (Latina, Ocular Instruments, Bellevue, WA, USA) (by U.Y.). By 0.1 mJ increments, the initial energy/pulse of 0.7 mJ was adjusted to the point that would induce a cavitation bubble and then kept constant throughout the procedure. Half an hour before and just after the SLT application, apraclonidine 1% was administered to prevent IOP spikes. No additional topical steroid or nonsteroid anti-inflammatory medication was prescribed for the postlaser period. Along with the generic name of the timolol maleate 0.5% combination used before the laser treatment (180° or 360°), the total number of laser spots and total energy exposure were also recorded.

Timolol maleate 0.5% combination therapy was discontinued on the day of treatment for the eye to be operated on, whereas ongoing therapy was not interrupted in the contralateral eye. Patients were examined for IOP elevation and anterior chamber reaction 1 hour and 1 day after intervention. Follow-up visits were scheduled for 1 week, 1 month, 3 months, and 6 months after the operation, during which the IOP of both eyes were evaluated with Goldmann applanation tonometry and any possible complications were noted and treated appropriately. IOP measurements were taken between 10:00 AM and 3:00 PM.

Timolol maleate 0.5% preparations were administered as one drop, once daily in the evening (8:00 PM) for FCs containing bimatoprost 0.03%, travoprost 0.004%, and latanoprost 0.005% and as one drop, twice daily (8:00 AM and 8:00 PM) for dorzolamide hydrochloride 2%, brinzolamide 1%, and brimonidine tartrate 0.2% combinations.

All data were analyzed using SPSS version 19.0 (SPSS Inc., Chicago, IL, USA). Demographic features including age, gender, operated eye (right or left), number of antiglaucoma medications used before laser therapy (FCs are considered as 2 drugs), distribution of FCs of timolol maleate 0.5%, number of laser spots, and total energy applied were described with mean, standard deviation (mean ± standard deviation) and/or frequency, percentage values, and 95% confidence interval. Mean values of repeated IOP measurements were displayed on a plot as a function of time. Tukey’s test along with analysis of variance (ANOVA) was used to correct for multiple comparisons of age, repeated IOP measurements and prelaser number of antiglaucoma medications among groups. Number of laser spots and total energy exposure between SLT groups were compared with Student’s t test. Chi-square test was used for multiple comparisons of categorical variables such as gender and operated eye. Appropriate p values of significance are displayed on the relevant graphs or the tables.

## RESULTS

A total of 40 patients were included in the study. There were 18 eyes in the SLT 180° group and 22 eyes in the SLT 360° group, along with 40 fellow-control eyes retained throughout the study. All subjects were Caucasian.

Table 1 shows the demographics and baseline characteristics of the patients. There were no statistically significant differences between the SLT 180° and SLT 360° treatment subgroups regarding age, gender, side, prelaser mean IOP, or prelaser number of antiglaucoma medications (p=0.986, 0.960, 0.817, 0.667, 0.696, respectively). However, number of laser spots along with total energy exposure were significantly different between the subgroups, as would be expected (p<0.001).

Table 2 shows the distribution of FCs of timolol maleate 0.5% used before the intervention between the SLT 180° and SLT 360° subgroups.

**Figure 1:**
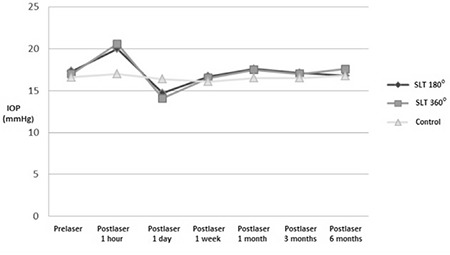
Course of mean intraocular pressures through six months SLT: Selective laser trabeculoplasty, IOP: Intraocular pressure

The mean IOPs in the SLT 180°, SLT 360° and control groups before laser and 1 hour, 1 day, 1 week, 1 month, 3 months, and 6 months after are shown in Table 3. There were no statistically significant differences among the groups with exception of postlaser 1 hour and postlaser 1 day (p<0.001 and p=0.010, respectively). Multiple comparison analysis with Tukey post hoc test showed significantly higher IOP in both the SLT 180° and SLT 360° subgroups compared to their controls at postlaser 1 hour (p=0.007, p<0.001) but significantly lower IOP only in SLT 360° subgroup compared to the controls at postlaser day 1 (p=0.013). Figure 1 shows the changes in mean IOP over time for the first 6 months. Accordingly, mean IOPs after SLT 180° and SLT 360° spiked remarkably at postlaser 1 hour and traced a slight trough at postlaser 1 day. However, no eyes had an IOP ≥30 mmHg or complications other than mild anterior chamber cells and flare (postlaser 1 hour) at any time. Moreover, there was no significant difference between repeated mean IOPs of control group (intraclass) through 6 months (p=0.191, ANOVA).

## DISCUSSION

The SLT/Med study, which was a prospective, randomized, multicenter clinical trial evaluating SLT vs. prostaglandin therapy as an initial treatment option demonstrated mean IOP reductions of 26.4% and 27.8%, respectively, from baseline.^2^ Lai et al.^21^ with regards to SLT vs. medical therapy reported mean IOP reduction of 32.1% and 33.2% from baseline at the 5 year follow-up, whereas reduction rates were statistically insignificant between 4-6 months in a comparison of SLT 360° to latanoprost 0.005% by Nagar et al.^26,27^ Two other prospective, nonrandomized studies by Melamed et al.^20^ and McIlraith et al.^28^ reported similar reductions from baseline with SLT as initial therapy. A retrospective study by Kara et al.^29^ reported mean reduction of 22.5% in IOP at 1 year. On the other hand, the meta-analysis by Cheng et al.^10^ evaluated 41 randomized trials and reported IOP reductions with timolol maleate 0.5% FCs that are comparable to the SLT trials mentioned above. Our results show that in patients receiving FCs, SLT may successfully sustain the same IOP levels at least for 6 months, which was consistent with a reduction in number of antiglaucoma medications by a mean of 2 at 6 months reported by Francis et al.^25^

With respect to safety, despite apraclonidine 1% administration for preventing IOP spikes, mean IOPs at postlaser 1 hour were significantly higher than contralateral control eyes in the study. Without prophylaxis, Helvacioglu et al.^30^ reported IOP spikes of 3-4 mmHg in almost all eyes at 1 and 2 hours postlaser. Our finding, however, is consistent with the previous reports wherein IOP spikes of 3-5 mmHg were detected in 8.4-10.3% of the subjects at 1 and 2 hours postlaser after prophylactic apraclonidine 0.5% or 1% administration.^24,31,32^ In addition, mean IOP course over 6 months revealed that SLT 180° and 360° achieved lower mean IOPs than that of the control group only at postlaser 1 day, with a statistically significant difference for SLT 360°. It should be noted that we did not set a washout period of 2-3 weeks before and discontinued the medications immediately after the laser procedure. Therefore, we attribute those lower IOPs at postlaser 1 day to the additive but not immediate IOP-reducing effect of SLT. Prophylactic apraclonidine 0.5% use just before SLT may also have reduced IOP additionally by a sustained effect.

The superiority of SLT 360° over SLT 180° in IOP-reducing efficacy is controversial. In patients with POAG, Nagar et al.^33^ reported no statistically significant difference between SLT 180° and SLT 360°, while SLT 90° produced the least effective outcome. However, studies by Shibata et al.^34^ and Prasad et al.^19^ suggest that SLT 360° is more effective in achieving lower mean IOPs or more limited IOP fluctuations than with SLT 180°. Moreover, Song et al.^35^ reported higher failure rates with SLT 180°. In our study, SLT 360° did not display a significantly higher IOP reduction over SLT 180° through 6 months.

A number of limitations should be kept in mind in the interpretation of our results. First, performing SLT on one eye and continuing the AGM therapy on the contralateral one may be associated with crossover effects for both treatment modalities. With SLT, McIlraith et al.^28^ displayed approximately 10% reduction of IOP in the untreated contralateral eyes for up to 6 months. Similarly, on the medication arm, The Ocular Hypertension Treatment Study (OHTS) showed 5.8% - 12% reduction of IOP in the untreated eyes as a contralateral effect of topical ß-blockers.^36^ However, we did not observe any findings in IOP suggesting crossover effects. Here we just speculate that crossover effects of AGM and SLT may either be masked or cancelling each other out in our study.

Measuring IOP during the daytime and only once per day in our study precludes drawing any conclusions about diurnal fluctuations or peak IOP levels. As diurnal fluctuation is an independent risk factor for progression of glaucoma,^37^ before proposing as a primary therapy, SLT should be shown to decrease the fluctuation to some extent as medications do. With respect to that, Nagar et al.^26^ reported success rates in fluctuation reduction as 50% for SLT and 83% for latanoprost.

The third and the most important limitation of this study was the assumption that all FCs reduce IOP similarly within a non-inferiority margin which indeed may not be the case. To our knowledge, there is no single clinical study comparing the efficacies of all beta-blocker timolol 0.5% combinations in IOP reduction. According to conclusions drawn from comprehensive review and meta-analysis manuscripts, prostaglandin-timolol FCs are likely to achieve better reduction in IOP than the other timolol combinations.^10,38,39^ As a recent systematic review concludes, bimatoprost/timolol FC in particular seems to achieve better reduction of IOP compared to other prostaglandin-timolol combinations containing latanoprost or travoprost.^40^ A clinical trial comparing all FCs to each other in terms of IOP reduction rate is therefore required to establish a reliable foundation.

In conclusion, FCs have provided improvement in patient compliance, reduction in level of preservatives, and thereby gained more preference recently. Alternatively, as our results suggest, SLT offers promising potential as a substitute for AGM equivalent to efficacy of FCs. One plausible argument remaining against SLT may be its diminishing effect over time.^23^ However, Avery et al.^41^ and Hong et al.^42^ showed safe and similar IOP reduction rates for repeat SLT comparable to first treatment. In this case, SLT may even indicate longer AGM-free periods for certain patients lacking compliance and suffering from preservative-related side effects. Further prospective studies that follow more patients for longer durations will be necessary before reaching a definitive conclusion in the comparison of FCs and SLT.

## CONCLUSION

SLT offers promising potential as a substitute equivalent to efficacy of FCs with timolol. However, SLT 360° may not achieve additional IOP reduction.

## Figures and Tables

**Table 1:**
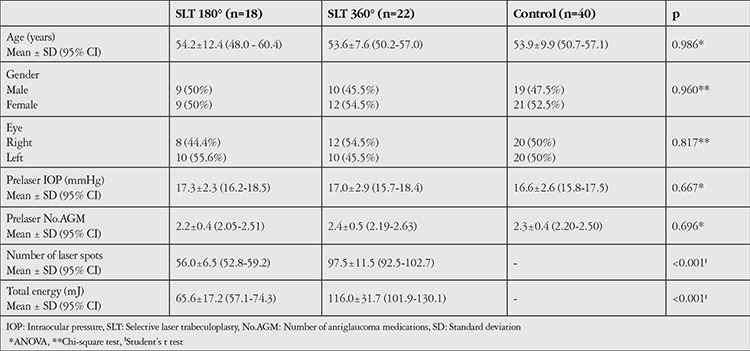
Demographic data, pretreatment values, and treatment features of the study participants

**Table 2:**
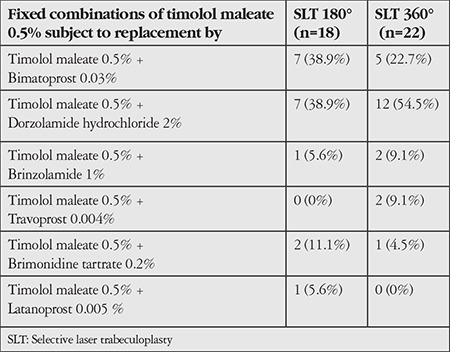
Distribution of fixed combinations with timolol maleate 0.5% used before the intervention between the treatment subgroups

**Table 3:**
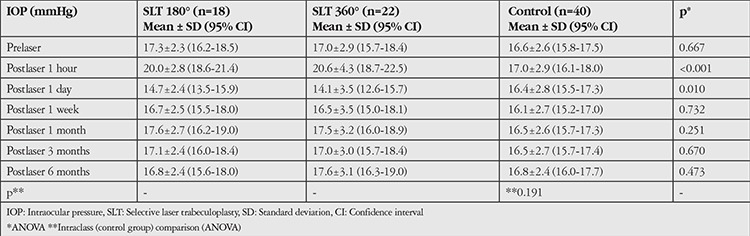
Mean intraocular pressures before and after Intervention

## References

[1] Quigley HA, Broman AT (2006). The number of people with glaucoma worldwide in 2010 and 2020. Br J Ophthalmol..

[2] Katz LJ, Steinmann WC, Kabir A, Molineaux J, Wizov SS, Marcellino G, SLT/Med Study Group (2012). Selective laser trabeculoplasty versus medical therapy as initial treatment of glaucoma: a prospective, randomized trial. J Glaucoma..

[3] Kass MA, Gordon M, Morley RE, Meltzer DW, Goldberg JJ (1987). Compliance with topical timolol treatment. Am J Ophthalmol..

[4] Schwartz GF, Reardon G, Mozaffari E (2004). Persistency with latanoprost or timolol in primary open- angle glaucoma suspects. Am J Ophthalmol..

[5] Rein DB, Zhang P, Wirth KE, Lee PP, Hoerger TJ, McCall N, Klein R, Tielsch JM, Vijan S, Saaddine J (2006). The economic burden of major adult visual disorders in the United States. Arch Ophthalmol..

[6] Realini T (2013). Selective laser trabeculoplasty for the management of open-angle glaucoma in St. Lucia. JAMA Ophthalmol..

[7] Hattenhauer MG, Johnson DH, Ing HH, Herman DC, Hodge DO, Yawn BP, Butterfield LC, Gray DT (1998). The probability of blindness from open-angle glaucoma. Ophthalmology..

[8] Fechtner RD, Realini T (2004). Fixed combinations of topical glaucoma medications. Curr Opin Ophthalmol..

[9] Khouri AS, Realini T, Fechtner RD (200). Use of fixed-dose combination drugs for the treatment of glaucoma. Drugs Aging..

[10] Cheng JW, Cheng SW, Gao LD, Lu GC, Wei RL (2012). Intraocular Pressure-Lowering Effects of Commonly Used Fixed-Combination Drugs with Timolol: A Systematic Review and Meta-Analysis. PLoS One..

[11] Latina MA, De Leon JM (2005). Selective laser trabeculoplasty. Ophthalmol Clin North Am..

[12] Wong MO, Lee JW, Choy BN, Chan JC, Lai JS (2015). Systematic review and meta-analysis on the efficacy of selective laser trabeculoplasty in open-angle glaucoma. Surv Ophthalmol..

[13] Samples JR, Singh K, Lin SC, Francis BA, Hodapp E, Jampel HD, Smith SD (2011). Laser trabeculoplasty for open-angle glaucoma: a report by the American Academy of Ophthalmology. Ophthalmology..

[14] Stein JD, Challa P (2007). Mechanisms of action and efficacy of argon laser trabeculoplasty and selective laser trabeculoplasty. Curr Opin Ophthalmol..

[15] Ruddat MS, Alexander JR, Samples JR (1989). Early changes in trabecular metalloproteinase mRNA levels in response to laser trabeculoplasty are induced by media borne factors. Invest Ophthalmol Vis Sci..

[16] Kramer TR, Noecker RJ (2001). Comparison of the morphologic changes after selective laser trabeculoplasty and argon laser trabeculoplasty in human eye bank eyes. Ophthalmology..

[17] Lee R, Hutnik CM (2006). Projected cost comparison of selective laser trabeculoplasty versus glaucoma medication in the Ontario Health Insurance Plan. Can J Ophthalmol..

[18] Russo V, Barone A, Cosma A, Stella A, Delle Noci N (2009). Selective laser trabeculoplasty versus argon laser trabeculoplasty in patients with uncontrolled open-angle glaucoma. Eur J Ophthalmol..

[19] Prasad N, Murthy S, Dagianis JJ, Latina MA (2009). A comparison of the intervisit intraocular pressure fluctuation after 180 and 360 degrees of selective laser trabeculoplasty (SLT) as a primary therapy in primary open angle glaucoma and ocular hypertension. J Glaucoma..

[20] Melamed S, Ben Simon GJ, Levkovitch VH (2003). Selective laser trabeculoplasty as primary treatment for open- angle glaucoma: a prospective, non-randomized pilot study. Arch Ophthalmol..

[21] Lai JS, Chua JK, Tham CC, Lam DS (2004). Five-year follow up of selective laser trabeculoplasty in Chinese eyes. Clin Experiment Ophthalmol..

[22] Mahdy MA (2008). Efficacy and safety of selective laser trabeculoplasty as a primary procedure for controlling intraocular pressure in primary open angle glaucoma and ocular hypertensive patients. Sultan Quaboos Univ Med J..

[23] Juzych MS, Chopra V, Banitt MR, Hughes BA, Kim C, Goulas M, Shin DH (2004). Comparison of long-term outcomes of selective laser trabeculoplasty versus argon laser trabeculoplasty in open-angle glaucoma. Ophthalmology..

[24] Grachner T (2002). Intraocular pressure response of capsular glaucoma and primary open-angle glaucoma to selective Nd: Yag laser trabeculoplasty: a prospective, comparative clinical trial. Eur J Ophthalmol..

[25] Francis BA, Ianchulev T, Schofield JK, Minckler DS (2005). Selective Laser Trabeculoplasty as a Replacement for medical Therapy in Open-Angle Glaucoma. Am J Ophthalmol..

[26] Nagar M, Luhishi E, Shah N (2009). Intraocular pressure control and fluctuation: the effect of treatment with selective laser trabeculoplasty. Br J Ophthalmol..

[27] McAlinden C (2014). Selective laser trabecuplasty (SLT) vs other treatment modalities for glaucoma: systematic review. Eye (Lond)..

[28] McIlraith I, Strasfeld M, Colev G, Hutnik CM (2006). Selective laser trabeculoplasty as initial and adjunctive therapy for open angle glaucoma. J Glaucoma..

[29] Kara N, Altinkaynak H, Satana B, Altan C, Yuksel K, Demirok A, Yilmaz OF (2011). Evaluation of factors influencing the outcomes of selective laser trabeculoplasty in primary open-angle glaucoma. Turk J Ophthalmol..

[30] Helvacioglu F, Akingol Z, Sencan S, Tunc Z (2014). Early intraocular pressure spikes observed after selective laser trabeculoplasty treatment and risk factors. Turk J Ophthalmol..

[31] Damji KF, Bovell AM, Hodge WG, Rock W, Shah K, Buhrmann R, Pan YI (2006). Selective laser trabeculoplasty vs. argon laser trabeculoplasty: results from a 1-year randomised clinical trial. Br J Ophthalmol..

[32] Grachner T (2001). Intraocular pressure response to selective laser trabeculoplasty in the treatment of primary open-angle glaucoma. Ophthalmologica..

[33] Nagar M, Ogunyomade A, O’Brart DP, Howes F, Marshall J (2005). A randomised, prospective study comparing selective laser trabeculoplasty with latanoprost for the control of intraocular pressure in ocular hypertension and open angle glaucoma. Br J Ophthalmol..

[34] Shibata M, Sugiyama T, Ishida O, Ueki M, Kojima S, Okuda T, Ikeda T (2012). Clinical results of Selective Laser Trabeculoplasty in Open-Angle Glaucoma in Japanese Eyes: Comparison of 180 Degree With 360 Degree SLT. J Glaucoma..

[35] Song J, Lee PP, Epstein DL, Stinnett SS, Herndon LW, Asrani SG, Allingham RR, Challa P (2005). High failure rate associated with 180 degree selective laser trabeculoplasty. J Glaucoma..

[36] Piltz J, Gross R, Shin DH, Beiser JA, Dorr DA, Kass MA, Gordon MO (2000). Contralateral effect of topial beta-adrenergic antagonists in initial one-eyed trials in the ocular hypertension treatment study. Am J Ophthalmol..

[37] Asrani S, Zeimer R, Wilensky J, Gieser D, Vitale S, Lindenmuth K (2000). Large diurnal fluctuations in intraocular pressure are an independent risk factor in patients with glaucoma. J Glaucoma..

[38] Hommer A, Sperl P, Resch H, Popa-Cherecheanu A, Qiao C, Schmetterer L, Garhöfer G (2012). A double masked randomized crossover study comparing the effect of latanoprost/timolol and brimonidine/timolol fixed combination on Intraocular pressure and ocular blood flow in patients with primary open-angle galucoma or ocular hypertension. J Ocul Pharmacol Ther..

[39] Ozer MA, Acar M, Yildirim C (2014). Intraocular pressure-lowering effects of commonly used fixed combination drugs with timolol in the management of primary open-angle glaucoma. Int J Ophthalmol..

[40] Lou H, Wang H, Zong Y, Cheng JW, Wei RL (2015). Efficacy and Tolerability of prostaglandin-Timolol Fixed Combinations: An updated systematic review and meta analysis. Curr Med Res Opin..

[41] Avery N, Ang GS, Nicholas S, Wells A (2013). Repeatability of primary selective laser trabeculoplasty in patients with primary open-angle glaucoma. Int Ophthalmol..

[42] Hong BK, Winer JC, Martone JF, Wand M, Altman B, Shields B (2009). Repeat selective laser trabeculoplasty. J Glaucoma..

